# Editorial: Environmental and agronomic factors affecting the chemical composition and biological activity of cannabis extracts

**DOI:** 10.3389/fpls.2024.1407262

**Published:** 2024-04-17

**Authors:** Nunzio Fiorentino, Carmen Formisano, Sebastiano Delfine, Giuseppina Chianese

**Affiliations:** ^1^ Department of Agricultural Sciences, University of Naples Federico II, Portici, Italy; ^2^ Department of Pharmacy, School of Medicine and Surgery, University of Naples Federico II, Naples, Italy; ^3^ Department of Agriculture, Environmental and Food Science, University of Molise, Campobasso, Italy

**Keywords:** cannabis extract, phytocannabinoid, agronomic practices, biostimulants, environmental stressors

Environmental and agronomic factors are paramount in shaping the chemical composition and biological activity of cannabis extracts. The plant is chemically complex, containing numerous phytocannabinoids, terpenes, non-cannabinoid phenols, flavonoids, alkaloids, and other secondary metabolites (Hanuš et al., 2016). Environmental stressors, influenced by agronomic factors, can induce chemical variability in the plant, which may be controlled and optimized for reproducible drug quality.

The cultivation environment, encompassing variables such as temperature, humidity, light intensity, and soil composition, profoundly influences the growth and development of cannabis plants (Danziger and Bernstein, 2021). Fluctuations in these environmental conditions can lead to significant alterations in the levels of cannabinoids, terpenes, and other bioactive compounds present in the plant (Akram et al., 2021; García-Tejero et al., 2014). Agronomic practices, including irrigation, fertilization, and pest management, also exert an impact on the chemical profile of cannabis extracts. For instance, the choice of nitrogen source and its ratio with other nutrients can significantly affect cannabinoid and terpenoid concentrations (Saloner and Bernstein, 2021). Moreover, the use of organic or mineral fertilizers, as well as the application of biostimulants, can modulate the metabolic pathways of the plant, resulting in changes in chemical composition (Di Mola et al., 2021).

On this important Research Topic, the guest editors made a call for original research articles and reviews through Frontiers in Plant Science. This Research Topic aimed to gather manuscripts on the phytochemical profiling of fiber hemp and seed oil, investigating variations in the chemical composition of phytocannabinoids and other secondary metabolites due to agronomic and environmental stressors. Specifically, guest editors encouraged scientists to submit their manuscript regarding crucial aspects related to studies focused on agronomic management, metabolomics investigations, extraction and isolation of secondary metabolites, bioactivity evaluation, and elucidation of the mechanism of action.

In this Research Topic 7 manuscripts were accepted for publication following peer-review.

The study conducted by Formisano et al. investigate the response of hemp plants to saline irrigation and plant-based biostimulant application. They find that saline irrigation significantly affects biomass yield and phytocannabinoid composition, with higher salinity levels leading to increased cannabidiol (CBD) predominance. Moreover, the application of a plant-based biostimulant mitigates the detrimental effects of saline irrigation on plant growth and nitrogen uptake, suggesting its potential for hemp cultivation in marginal soils due to salinization. Massuela et al. investigate the impact of nutrient stress at flowering stage on biomass, CBD yield, and nutrient use efficiency of medicinal cannabis. They find that nutrient-deprived plants show lower inflorescence yield but higher CBD concentration, leading to a more sustainable use of fertilizers. The study highlights the importance of optimizing fertilization regimes for maximizing CBD yield while reducing the environmental footprint of the cropping cycle. Garrido et al. explore the effects of exogenously applied signaling molecules on cannabinoid accumulation in medical cannabis plants. They find that foliar application of certain signaling molecules increases cannabinoid content in leaves and inflorescences, suggesting a potential method for modifying the chemical profiles of medical cannabis to enhance its pharmacological properties. Fernández et al. emphasize the importance of characterizing cannabis according to its metabolome, beyond its cannabinoid profile. Through metabolomic analyses of different cannabis varieties, the study identifies molecular markers associated with fungal infection and highlights variations in metabolomic profiles even within the same variety. This approach provides valuable insights into crop health and helps in understanding the physiological effects of cannabis beyond its primary cannabinoids. Islam et al. investigate the effects of LED spectral changes on reactive oxygen species (ROS) and cannabinoid accumulation in hemp plants. They find that ROS metabolism plays a crucial role in morpho-physiological acclimation and cannabinoid accumulation, with higher ROS levels leading to increased cannabinoid production.

Malík et al. investigate the impact of amino acid-based biostimulants application on the cannabinoid and terpene profiles of medical cannabis plants, along with different nutritional cycles, and found that the amino acid supplement significantly influenced the tissue ionome of the plants. Interestingly, the recirculation cycle led to higher nitrogen and sulfur accumulation in plant tissues but lower calcium content compared to the “drain to waste” cycle. Additionally, the study observed a shorter maturation period for plants in the “drain to waste” cycle but lower cannabinoid yields. The amino acid supplements also reduced cannabinolic acid content but increased monoterpenes, such as limonene and β-myrcene. Overall, the research highlights the sensitivity of cannabinoid and terpene metabolism to nutrient supplies, emphasizing the advantages and disadvantages of different fertilization approaches and biostimulants.

Saloner and Bernstein study the impact of different nitrogen sources on cannabis plant function and metabolite production. They find that the ratio of ammonium (NH_4_^+^) to nitrate (NO_3_^-^) significantly affects cannabinoid and terpenoid concentrations, with moderate levels of NH_4_^+^ enhancing production without damaging plant function. However, high NH_4_^+^/NO_3_^-^ ratios are not recommended due to potential toxicity damage.

In general, the manuscripts selected for this Research Topic significantly contribute to shed light on various aspects of the intricate relationship between environmental and agronomic factors and the phytochemical composition of cannabis extracts. The findings reported in this Research Topic serves as a valuable resource for researchers and farmers involved in the cannabis and hemp industry, providing evidence-based strategies for optimizing crop management practices and harnessing the full potential of this versatile plant. The researches highlight the potentiality of a careful agronomic management and environmental control in optimizing the chemical profile of cannabis extracts for medicinal and industrial purposes. By gaining insights into how factors such as irrigation, fertilization, and light spectrum affect cannabinoid and terpenoid concentrations, cultivators can develop more efficient and sustainable cultivation practices. Moreover, the insights in the application of novel interventions, such as biostimulants and signaling molecules, in enhancing cannabinoid production and modulating plant responses to environmental stressors, pave the way for future research aimed at maximizing the yield and quality of cannabis extracts while minimizing environmental impact.

## Author contributions

NF: Writing – review & editing. CF: Writing – review & editing. SD: Writing – review & editing. GC: Writing – original draft, Writing – review & editing. GC: Supervision, Funding acquisition, Writing – original draft, Writing – review & editing.

## References

[B1] Akram N. A. Shafiq F. Ashraf M. Iqbal M. Ahmad P. (2021). Advances in salt tolerance of some major fiber crops through classical and advanced biotechnological tools: A Review. J. Plant Growth Regu. 40, 891–905. doi: 10.1007/s00344-020-10158-5

[B2] Danziger N. Bernstein N. (2021). Light matters: effect of light spectra on cannabinoid profile and plant development of medicinal cannabis (Cannabis sativa L.). Ind. Crops Products. 194, 113351. doi: 10.1016/j.indcrop.2021.113351

[B3] Di Mola I. Conti S. Cozzolino E. Melchionna G. Ottaiano L. Testa A.. (2021). Plant-based protein hydrolysate improves salinity tolerance in Hemp: agronomical and physiological aspects. Agronomy 11, 342. doi: 10.3390/agronomy11020342

[B4] García-Tejero I. F. Durán-Zuazo V. H. Pérez-Álvarez R. Hernández A. Casano S. Morón M.. (2014). Impact of plant density and irrigation on yield of hemp (*Cannabis sativa* L.) in a mediterranean semi-arid environment. J. Agric. Sci. Technol. 16, 887–895.

[B5] Hanuš L. O. Meyer S. M. Muñoz E. Taglialatela-Scafati O. Appendino G. (2016). Phytocannabinoids: a unified critical inventory. Nat. Prod. Rep. 33, 1357–1392. doi: 10.1039/C6NP00074F 27722705

[B6] Saloner A. Bernstein N. (2021). Nitrogen supply affects cannabinoid and terpenoid profile in medical cannabis (Cannabis sativa L.). Ind. Crops Products 167, 113516. doi: 10.1016/j.indcrop.2021.113516

